# Case and Observations in Surgery at the Paris Hospitals

**Published:** 1854-03

**Authors:** E. Williams


### Art. II.—CASES AND OBSERVATIONS IN SURGERY AT THE PARIS HOSPITALS.

BY E. WILLIAMS, M. D., IN A LETTER TO PROFESSOR GROSS.

### Case 1.—*Burn of the Eyes by Lime.*

On the 27th of August, I saw a man at the eye clinic of M. Désmarres, who, two days previously, had fallen with his face in a vessel of slaked lime. He stated that he immediately felt an intense burning pain in both eyes and ceased entirely to see. M. Désmarres removed from the palpebral sinuses a considerable quantity of lime mixed with coagulated blood. The corneæ, seemingly only superficially cauterized, were still partially transparent, so that the pupil and iris were visible through them—the right being more seriously involved than the left. The surrounding conjunctiva and schlerotica were of a dirty, yellowish white color, and completely insensible to the contact of the finger or an instrument. The patient was free from fever; there was no photophobia,—in short, he did not suffer the slightest pain, but could not see to walk. This condition of freedom from suffering and inflammatory action lasted for *two weeks.* On the 14th day, the right eye, which was most deeply burned, was still unchanged; but the left was red and painful, with considerable chemosis, and there was a semilunar ulceration of the cornea near its border, occupying about one-fourth of its circumference. By means, however, of the most energetic antiphlogistic measures, with repeated scarifications of the vessels of the globe around the cornea, this eye was saved, and the patient now sees sufficiently to transact business. But with the right organ the result was very different. On the 15th or 16th day, intense inflammation was developed. The surface of the cornea and the whole extent of the conjunctiva, deprived of vitality by the lime, was sloughed; and, in a very few days, firm adhesions occurred between the lids and the globe of the eye, in spite of every effort which was made to prevent it. From day to day the cicatricial tissue, which took the place of the conjunctiva, continued to contract from behind forwards, till it had produced the most complete symblepharon. The cornea, transformed into a cicatrix, contracted from the circumference towards the centre, and presented itself as a small, hard, fungous excrescence, which protruded between the edges of the lids at the central part. I saw this patient yesterday, and found him in the same condition, excepting that the opacity of the left cornea is gradually disappearing, and he sees better with it than he did some weeks ago.

At the first sight of this unfortunate patient, M. DÉSMARRES made a very unfavorable prognosis—stating that the right eye would be irretrievably lost, but the other might possibly be saved —that although no violent symptoms were then present, the *absence* of *sensibility* in the right cornea and conjunctiva was sufficient evidence that they would be, sooner or later, eliminated in the form of an eschar and followed by symblepharon. His experience had proved to him that the commencement of the eliminating process in burns of the eye, does not occur before the fifteenth or twentieth day after the accident, and that it was hazardous to base a favorable prognosis, at an *early period*, upon the apparently *slight character* of the burn. In indicating the great reserve which should characterize the conduct of the surgeon in this respect, he related the history of a young woman whom he treated some three years ago, for a similar accident produced by sulphuric acid, that her jealous husband, in a fit of passion, had dashed in her face. Both corneæ, when he first saw her, were still almost perfectly transparent, but their surfaces, with that of the surrounding conjunctivæ, were completely *deprived of tactile sensibility*. The woman had no pain or fever, and continued to see moderately well. *Twelve days* passed before severe inflammation was developed. Extensive sloughing of the cornea and schlerotica then occurred in the left eye, the crystalline lens escaped, and, in less than one month from that time, the ocular and palpebral surfaces of the conjunctiva were solidly agglutinated together. Subsequent but unsuccessful attempts were made to separate them and insert an artificial eye, and the patient remains with an incurable deformity. Fortunately the left eye, less profoundly cauterized by the acid, was saved, after running the most imminent danger. There is now in the Hospital of the Faculty a young man who, seven years ago, received some quick lime in the eye. It was immediately washed out with a stream of cold water, and a constant application of the same fluid, kept up for several days, prevented him from suffering a great deal; but at the end of three weeks, there was symblepharon, with union also of the free edges of the lids with each other. He came into the hospital some weeks ago, requesting an operation to remedy the deformity. From the prominence of the organ it was evident that there was a staphyloma behind the agglutinated lids. On the 7th of December, M. Nelaton separated, with a bistoury, the edges of the lids, dissected them loose from the balls, and then excised a portion of the staphyloma, hoping to prevent a re-union, and enable the patient to wear an artificial eye. High inflammation followed the operation, and the adhesion was again re-produced, excepting the free edges of the lids, which still remain separated. He now proposes to detach them again and immediately insert an artificial eye, and retain it constantly there so as, if possible, to prevent the reunion. But the use of this means, or of any other foreign body inserted between the lids and ball, often creates excessive inflammation; or, even if it is tolerated by the patient, the union gradually occurring at the bottom of the conjunctival cul de sac, pushes the foreign body forward with the irresistable force of cicatricial contraction, and expels it from the cavity, and agglutination is inevitable. Again, should the surgeon succeed in maintaining this substance between the cicatrizing surface for many months, and then remove it, the cavity will then be almost surely obliterated. Mr. N. remarked that in a case of a similar kind, he once operated, and made the patient wear a thin plate of metal for six months, then removed it, and, in a short time, the adherence was reproduced. Every one who knows the incontrollable force with which a cicatrix contracts, can readily conceive the almost entire uselessness of all means usually adopted to prevent adhesions when the whole extent of the conjunctival surfaces has been involved. When, however, the bottom of the cul de sac has remained intact—when the union is limited to the anterior part—then the chance of success by the use of the metallic plate or enamel eye interposed, is much greater. I remember having seen M. VELPEAN operate upon a case of symblepharon last summer, in which he passed a needle with a strong thread through the middle of each lid, and attached them, the one over the forehead, and the other beneath the chin, so as to separate them from all contact with the globe. This was kept up for several weeks, but finally the surfaces were fatally soldered together as before. You remember, doubtless, the little boy from Indiana, with the same deformity produced by the explosion of gun powder, upon whom you operated in my presence in the spring of 1852, and in whom the difficulty was again reproduced.

Two other procedures have been suggested and practiced in this almost irremediable deformity. The one dissects up the adhesions, inverts the lids, and maintains them thus with the cuticle in contact with the globe, till cicatrization is completed. The other divides the lids perpendicularly at their middle points, detaches the flaps from the globe beyond the ordinary limits of the conjunctiva, everts them completely, and keeps them thus during the cicatrizing process. This latter method is the only one which can be resorted to with much prospect of success, when the entire extent of the conjunctiva is adherent. After the cicatrization, the divided edges of each lid can be pared and readily united with needles and sutures.

In consequence perhaps of the rarity of burns of the eye, but little is said of them in treatises upon surgery, and hence the above observations may not be without interest to the general practitioner. During the past six months, I have followed assiduously the eye clinic of Désmarres, where are present, every other day, from fifty to one hundred and fifty patients, and the first case related is the only one of burn of the eye that I have seen. I have observed enough of this accident, however, to induce me in the future to use great reserve in the prognosis of such cases, however slight.

---

Case 2.—*Strangulated Femoral Hernia--Operation—Death by Cholera.*

On the 6th of December, a woman came into the hospital, stating that she was suffering from strangulated hernia. The rupture had existed for ten years, and she had worn a truss which did not keep it perfectly reduced. It caused no suffering or special inconvenience, however, till the 4th of December, when she was taken, without any appreciable cause, with vomiting, and soon after observed that the tumor in the groin was larger than usual, and somewhat painful to the touch. A physician was called, who, by long continued taxis, succeeded only in very slightly diminishing the size of the swelling without reducing it. On the following morning the vomiting still continued, and there had been no discharge from the bowels. Renewed efforts at taxis were made, but with no better success than the first. On Tuesday, at 12 o'clock, she entered the hospital, and, at 7 p. m., I examined her in company with the chief Intern of the hospital. The vomiting had then entirely ceased—her countenance tranquil, skin natural, pulse slow and soft—in short, she did not seem to be suffering at all. There had been, however, no discharge from the bowels since the previous Saturday. In the left groin was a tumor of the size of a small hen's egg, ovoidal in shape with the long diameter in the direction of Poupart's ligament, and which she said had not changed in size for three days. It seemed to mount up in front of the ligament, but, when carefully examined, a pedicle was felt extending from it beneath the ligament, and restraining thus the freedom of its movements. To the feel it was very hard, giving exactly the sensation of a hypertrophied lymphatic gland; it was entirely dull upon percussion, and but very slightly painful to the touch. It seemed impossible that a patient free from all suffering or fever, and with a countenance perfectly calm, should be laboring under strangulated hernia—an affection which transforms every feature into an index of intense suffering and anxiety. A purgative injection was ordered, which brought away some fœcal matter.

Wednesday morning, 8 o'clock, same tranquil state continues—pulse natural—no vomiting. Prof. NELATON prescribed a dose of castor oil, which was very soon vomited. In the afternoon, restlessness, vomiting and hiccups appeared, and some unsuccessful attempts at reduction were made.

Thursday morning, 8 o'clock, same symptoms continued and she felt some uneasiness in the region of the tumor, but firm pressure did not cause much pain. The previous existence of a hernia, the pedicle extending from the tumor into the abdomen, and the obstinate constipation, with vomiting, left little room for doubt about the nature of the difficulty. The injection had caused the discharge of some fœcal matter from the bowels; but it, doubtless, did not come from above the constriction. Nothing given by the mouth had traversed the whole canal. The taxis proving unsuccessful, Prof. N. resolved upon an operation. On cutting through the skin and superficial fascia, he came upon a fatty mass much resembling epiploon, but he incised it freely and at length perceived the bluish, semi-transparent sac. When it was opened a small jet of bloody serum issued, and the little knuckle of intestine, purple from the strangulation, presented itself. After incising freely the sac, he seized, with the fingers, the sides of the canal through which the intestine had protruded, and, making rather strong traction, notched in three places, with a probe-pointed bistoury, the superior constricted part. The intestine was then reduced without any difficulty, the vomiting ceased, and soon afterwards she had several free evacuations from the bowels. The wound was not closed by sutures, but simply dressed with charpie and a bandage. For the following forty-eight hours the case progressed very favorably, when all at once she wrs attacked with cholera. Cyanosis soon appeared, the voice was extinct, pulse gone, skin cold and shriveled, without the least tympanitis, pain upon pressure or other symptom of peritonitis. She died on the 13th, four days after the operation. At the post mortem, no trace of peritonitis were discovered; the knuckle of intestine that had been imprisoned was still purplish but not gangrenous. At the abdominal opening of the crural canal and extending slightly into the cavity of the abdomen, were three slight incisions involving only the peritoneum and eillular tissue beneath it.

There are several points worthy of special remark in the history of this case.

First—The complete subsidence of the restlessness and vomiting after they had existed for 24 hours, and that too without the reduction of the hernia—the length of time (nearly 48 hours) that this state of calmness and freedom from pain and vomiting lasted—and finally the reappearance of the grave symptoms and the consequent necessity of an operation.

Second—The remarkable hardness of the tumor, and its resemblance to an enlarged lymphatic gland—the complete dulness upon percussion although it contained a portion of intestine filled with gas, and its almost entire freedom from pain whether spontaneous or from pressure.

Third—The seat of the strangulation, that is the peritoneum simply at the superior orifice of the femeral canal. There was not the slightest constriction either at gimbernats ligament, the falciform process of the fascialata (Hay's ligament) or the opening where the hernia traversed the fascia cribriformis, the three points that are always accused as being the seat of the strangulation. In regard to this woman, Prof. Nélaton remarked that he had seen a similar case not long since, where the tumor was entirely free from pain. There were vomiting, obstinate constipation, cramp, great depression, and that peculiar anxious and contracted state of the features so characteristic of strangulation. The tumor was irreductible, yet all these symptoms, except of course the *constipation*, subsided, and for some 36 hours the patient was perfectly tranquil as in the case above cited. They at length returned with extreme violence, and he was compelled to operate and found a portion of intestine severely strangulated. The posibility of the disappearance of the urgent symptoms for a length of time, notwithstanding the persistence of the strangulation, and the entire freedom from pain in the region of the tumor, is a fact which should be faithfully borne in mind. PROF. NÉLATON's method of operating for strangulated hernia is exceedingly simple. He cuts without hesitation through all the tissues till he sees the bluish sac, rendered so by the sanguinolent serum and purplish color of the intestine which are seen through it; he then divides the sac carefully, and taking hold of it on each side makes sufficient traction to bring into view the strictured part, (which he says exists in the great majority of cases at some one of the little perforations in the cribriform fascia through which the hernia has protruded) and then notches it at several points so as to enable him to reduce the contents. There is certainly no danger in cutting freely through the skin and other tissues, whether fascia or adipose substance, till the purplish transparent sac is brought into view. The bluish color of the sac renders it unmistakable if its contents are *intestinal*, and if they are omentum, no great inconvenience arises if they are cut. Many young surgeons I am confident, allow patients to die without an operation, or else, defer it so long that there is no hope of success, from timidity originating out of the *classic idea* of the great difficulty and danger of the operation. As a general rule at least, it is certainly one of the simplest in all surgery.

The accumulation of fat in the subperitoneal cellular tissue present in this case, and giving rise to the *hardness* of the tumor and its complete dulness upon percussion, is often a great source of embarassment to the inexperienced operator. But with the idea present in his mind that this deposit, often quite thick, of adipose substance frequently exists and must necessarily be cut through before the sac is reached, he proceeds coolly but of course *prudently* in his operation. It is by thus thickening the walls of the tumor by the *deposit of fat* that nature sometimes obliterates the sac and effects a spontaneous cure. Or, the neck merely of the sac being obliterated after the reduction of the contents, there remains a small isolated serous cavity which may subsequently become the seat of a serous accumulation in the form of a cyst. I saw, a few days ago, in the wards of PROF. VELPEAU, a case of this kind. The woman had a hernia which had disapeared spontaneously, but, some months afterwards, a tumor began to be developed in the same point which increased slowly till it had acquired the volume of a hen's egg. He made a free incision into it, gave issue to the fluid it contained and filled the cavity with charpie so that it healed by granulation from the bottom. Cysts supposed to be developed in the lymphatic glands of the groin, have doubtless sometimes their origin in this source.

While on the subject of hernia, I must tell you of a couple of novel operations recently performed by M. MAISONNEUVE, who stands at the head of *progressive surgery* in Paris, and *progress* you know in *surgery* and *politics* is rarely effected without the *loss of blood.* The one was a patient upon whom he had operated for strangulated hernia, and in whom the symptoms of strangulation still continued. The other, a case in which the hernia had been reduced by the taxis but all the urgent symptoms still persisted. In both he opened the abdomen in the right lumbar region, searched a portion of the small intestine *above* the seat of the stricture which he determined by its distension ; then found the caecum, made an incision into each and united them by sutures so as to establish a route for the alimentary matters. Both patients were almost in *articulo mortis* when this proceedure was adopted, and of course *died.* Such an operation in such desperate circumstances, if not *advisable*, is at least *excusable*, particularly in Paris. I should like to give you a summary of the many operations of an *analagous nature*, that I have seen performed by this *origiual and enthusiastic* surgeon, but my space will not allow it. He has a perfect mania for hazardous and unheard of operations. Aside from this, there are many traits in his character that I admire very much, and many of his novel proceedures are exceedingly ingenious; besides, he operates most beautifully.

CASE 3.—*Ascites taken for a Cyst of the Ovary—Injection of Iodine—Death.*

Early in November, there entered the hospital a woman aged 31 years with an enormous distension of the abdomen. She stated that fifteen months previously, shortly after a natural accouchment, she perceived that her abdomen was enlarging, and, supposing herself pregnant, consulted her physician who told her it was a tumor. The enlargement increased very rapidly, and on the 12th October last, she was tapped in consequence of the extreme difficulty of breathing, and over *six gallons* of a clear citron colored liquid withdrawn. The swelling, however, commenced immediately, and in less than three weeks the abdomen was as large as before the puncture. On the 14th November, M. NÉLATON examined the patient and pronounced the tumor to be a *cyst of the ovary.* In his clinic he stated that the ancients possessed no physical means of diagnosing *ascites* from a cyst of the ovary when it had acquired a *great size and filled the entire abdominal cavity;* but that now the *sign* of Rostan renders it comparatively easy. In ascites the patient lies upon the back and the intestines distended with gas, float upon the liquid, and hence there is dulness upon percussion in the right and left lumbar regions with sonoriety in the umbilical. On the contrary, a cyst of the ovary mounts up in front of the intestines, pushes them to each side and gives rise to dulness in the *umlilical and sonoriety* in the *lumbar regions*, which was the case in the patient under consideration. There was one source of error, however, which, though extremely rare, might give rise to wrong diagnosis. If the intestines were perfectly free from gas they would not float upon the liquid; or again, the existence of *ancient adhesions* might bind them down and prevent them from rising to the surface. The rapidity with which the fluid accumulated, rendering tapping necessary every two or three weeks, demanded summary treatment, otherwise, the patient would die from exhaustion in a very few months. In this view of the subject, and encouraged by the reports of a number of cases of ovarian cysts cured by injections of the tincture of iodine, he resolved upon this treatment, but not without serious misgivings as to the consequences of injecting an irritating fluid into such a vast cavity. But the woman was still young, of a moderately good constitution, and the liquid previously withdrawn, was limpid, indicating a favorable surface for adhesive inflammation; besides, according to him, there was no evidence of serious organic lesion in any of the viscera. But, contrary to PROF. N's well known character for minute and careful examinaticns, he omitted the *heart* entirely. On the day previous to the operation, I examined, with the chief intern M. DOLBEAU, the chest of the woman and found the heart's actions very irregular and tumultuous, extensive dulness upon percussion in the precordial region, and a strong bellows murmur with the first sound. Besides this, there was slight edema of the inferior extremities.

On the 18th November, he punctured the abdomen, drew off an enormous quantity of clear serum and then injected about a quart of the following solution: Tinct. of iodine, one part; dist. water, two parts; iod. of potash, q. s.; to prevent the precipitation of the iodine. The injection remained about five minutes, and then was allowed to issue by the canula. It escaped, however, with difficulty and at least half was necessarily left in the abdomen. The woman immediately became faint, the lips pale, pulse feeble and frequent with much general anxiety; but she did not complain of the abdomen. During the following forty-eight hours she vomited frequently, had great thirst, cold skin, and a very rapid feeble pulse. Still there was no acute abdominal tenderness or other symptoms of intense peritonitis. After two days the pulse became fuller, the vomiting ceased, the lips redder, and expression of countenance much better.

Nov. 22—patient appears much better—no abdominal tenderness—no vomiting; but the fluid is very rapidly re-accumulating and there is now perhaps near two gallons in the abdomen. Desiring something to eat, a little soup with a small piece of chicken was allowed her. She seemed quite well till two o'clock the following morning, when she suddenly called for the nurse, complained of a severe pain in the head and died in a very few seconds.

Post mortem. On opening the abdomen, an old and very *consistent false membrane* was found binding the small intestines firmly against the vertebral column.* The collection of liquid was in the peritoneal cavity in *front* of the intestines, and hence the dulness in the umbilical and sonoriety in the lumbar regions. The ovaries were a little enlarged, and in one of them were several small cysts of the size of an ordinary pea.

Upon the surface of the peritoneum were some shreds of recent false membrane, the result of the slight peritonitis produced by the injection. The liver was cirrhosed. The pericardium was obliterated by adhesions, and there was enormous hypertrophy of the heart, with great narrowing and thickening of the mitral valve; a large clot existed in the right ventricle and the left auricle was greatly dilated, sufficiently in fact, to contain an adult fist.

The death in this case, nearly five days after the operation, and when all the symptoms of peritonitis had subsided, can not, I think, be attributed to the injection. The grave organic lesion of the heart, is sufficient explanation of the sudden termination of life in the midst of apparent improvement. But the error of diagnosis led to a treatment which *might have been* immediately fatal, and which this eminent surgeon would by no means have employed, if he had suspected an ascites produced by an organic disease of the heart. He was led into the mistake by his great confidence in the physical sign of Rostan, as afforded by percussion, although aware of the possibility of certain physical conditions that may give rise to the same phenomena even in ascites.

Several cases have recently been published in the French journals, of cysts of the ovary cured by repeated injections of iodine, but it is by no means certain that many of them were not ascites produced by chronic peritonitis, or other affections much more amenable to this treatment than ovarian dropsy. Still, as there are several well authenticated cases of cure upon record, it must be acknowledged to be a valuable resource in this almost irremediable affection. In 1840, Prof. Dieulafoy, of Toulouse, struck with the good results and comparative innocuity of injections of iodine into chronic abscesses, hydrocele, external cysts of various kinds, and even into the joints as practised by Velpeau, conceived and executed the idea of throwing the same substance into the peritoneum in cases of chronic peritonitis followed by intractable dropsy. His first patient, reduced by repeated tappings to the very brink of the grave, was completely cured after three injections at intervals of from two to four weeks; most probably by the complete obliteration of the peritoneal cavity by adhesive inflammation. This remarkable cure brought the treatment into vogue, and it has since been greatly popularized by VELPEAU and other distinguished surgeons. One thing is certain, that the contact of this substance with the peritoneum, thickened and altered as it is in chronic peritonitis and other affections does not often produce grave accidents. But inject the same substance into a healthy peritoneum and death is almost certain. A few months since I saw MR. MAISONNEUVE tap an ovarian cyst, and afterwards inject a large quantity of solution of the tincture of iodine, most of which could not be made to run out again. The woman was instantly seized with excruciating pains in the abdomen, sinking of the pulse, coldness of the surface and extremities, excessive anxiety of the countenance, and in *less than three hours she was dead.* On examination it was found that the canula had become deranged, and allowed the injection to enter the *peritoneal cavity.*

The array of experiments upon *dogs* in opposition to injections of iodine for ascites is of little weight; because, aside from the *difference of species*, there is a very wide difference also, between the healthy peritoneum of a dog and this membrane as it is thickened and altered by disease in the human subject. They are about as irrelavent to the question, as are the experiments of MALGAIGNE upon *rabbits* in order to determine the influence of the extended, or semiflexed position of the limb in the treatment of fractures! Between the size and direction of the muscles with respect to the bones, the shape of the joints, arrangement of the ligaments, &c., in the rabbit, and in the "lord of creation" there is as great a difference as there is between the *moral attribute* of these two animals. These questions can only be solved by trials upon the human subject, and thus far experience sanctions injection of iodine, under certain circumstances, into the peritoneal cavity, ovarian cysts, the pleura and even the joints. During the past summer I saw it thrown into the pleura by Valleix, with very decided benefit in a case of hydrothorax from chronic pleurisy, and several times have I seen VELPEAU inject the cavity of the knee joint with the same good results. He says he has injected the knee joint in thirty cases, and the phenomena which follow are precisely similar to those that are observed after the injection of a hydrocele,—slight pain, redness and swelling which subside entirely in three or four days. He alledges that this fluid injected into the joints, is not dangerous—that it cures a certain number of cases, especially when uncomplicated—that it assists other remedies in curing others—and that he has never seen it followed either by suppuration or anchylosis. Of course he only resorts to it in case of failure from ordinary and more simple treatment. The same procedure has also lately been proposed as a means of *dissolving* foreign bodies which form in the articulations. This substance, however, when thrown in considerable quantities into large cavities is liable to be absorbed and produce severe poisonous effects, especially, when it does not issue freely through the canula, which is often the case. On the 20th of June, I was present and saw PROF. NÉLATON inject a very large chronic abscess at the anterior part of the thigh, caused by caries of the spine. He threw up a pint and a half of fluid, at 10½ o'clock, and only about half of it could be got out. Three hours afterwards, the boy began to feel giddiness and dimness of vision, followed soon by vomiting and extreme "malaise." The skin was moist, extremities cold, pulse small and thread-like, respiration accelerated, great prostration of strength. This formidable array of symptoms lasted all night, and on the following morning, in addition, there were *cough*, *sore throat*, *extinction of the voice*, inarticulate moanings, and enormous swelling, with lividity of the upper eye lids. On the 22d, he seemed stronger, but still complained greatly of a pain in his throat which was dry but not otherwise altered from health. But the respiration, especially *inspiration*, was very laborious with hoarseness and a croupy cough. He was evidently in danger of suffocation from oedema of the glottis, and PROF. N. was on the point of resorting to tracheotomy, when under the influence of purgation, ice internally, sinapisms to extremities and blisters on each side of the larynx, he was relieved and in a few days all the symptoms disappeared.

I have several other cases upon my note book, of which I intended to have spoken, and especially of the operation of *urethotomy*, as recently proposed by M. RÉBARD in a very remarkable monograph upon strictures of the urethra. By means of an instrument of his own invention, he makes a very deep incision through the stricture from within outwards, so as to be able at once to insert the largest sized bougie of Bénéqué's scale. I have seen the operation performed several times with a modification of his instrument, a sort of lithotome caché,—but must defer giving you the results of my observations till some future occasion, as my letter is already much too long.

